# Obstruction caused by suprasellar cisterna arachnoid cyst expansion after ventriculoperitoneal shunt in children with hydrocephalus

**DOI:** 10.1002/ped4.70004

**Published:** 2025-04-30

**Authors:** Dapeng Li, Wenping Ma, Ming Ge, Di Zhang

**Affiliations:** ^1^ Department of Neurosurgery Beijing Children's Hospital Capital Medical University National Center for Children's Health Beijing China

**Keywords:** Secondary obstructive hydrocephalus, Suprasellar cisterna arachnoid cyst, Ventriculoperitoneal shunt, Ventriculostomy

## Abstract

**Introduction:**

Suprasellar cistern arachnoid cysts are rare non‐neoplastic cystic lesions that represent nearly 1% of all intracranial arachnoid cysts. Symptomatology can vary; small cysts typically do not produce any symptoms, whereas larger cysts may lead to headaches, blurred vision, and other related issues. However, the etiology, pathogenesis, and natural history of this condition remain unclear.

**Case presentation:**

This study includes four cases from our hospital from the year 2016 to 2021, first diagnosed with hydrocephalus received ventriculoperitoneal shunt treatments, the patients complicated with enlargement of the suprasellar cisterna arachnoid cyst occurred following ventriculoperitoneal shunt placement, leading to secondary obstructive hydrocephalus and received neuroendoscopic third ventriculostomy and cyst fenestration.

**Conclusion:**

Suprasellar cisterna arachnoid cysts may be related to intracranial pressure dynamics. Fluctuations in intracranial pressure can lead to variations in cyst size and may result in associated symptoms. Monitoring imaging and symptoms before, during, and after surgery, along with comprehensive follow‐up, is important.

## Graphical abstract

This article focuses on four cases of hydrocephalus after ventriculoperitoneal (VP) shunt in our center from 2016 to 2021. Imaging follow‐up showed that the secondary suprasellar cisterna arachnoid cyst was enlarged and then formed obstructive hydrocephalus. The clinical symptoms were relieved after a suprasellar cisterna cyst stomy. Combined with two cases of spontaneous regression of suprasellar cisterna arachnoid cysts reported in the literature, this article puts forward our own views on the etiology, pathogenesis, and natural history of suprasellar cisterna arachnoid cyst, to help the diagnosis and treatment of this disease.

We found suprasellar cisterna arachnoid cysts and secondary suprasellar cisterna cysts were found during the follow‐up after the VP shunt of hydrocephalus, which caused obstructive hydrocephalus and required a second surgical intervention. Brain magnetic resonance imaging, especially the application of a three‐dimensional steady‐state interference sequence for the diagnosis of suprasellar arachnoid cyst.

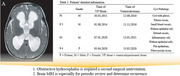

## INTRODUCTION

Intracranial arachnoid cysts account for approximately 1% of space‐occupying lesions, with suprasellar cistern cysts comprising 5%–12.5% of this group.^1^, ^2^ Although rare in clinical practice, they can cause obstructive hydrocephalus, significantly affecting children's quality of life.^3^, ^4^ Common complications include headaches, visual disturbances, hormonal imbalances, and hypopituitarism. Children with hydrocephalus who undergo ventriculoperitoneal (VP) shunting may develop secondary suprasellar arachnoid cysts that can progressively expand and lead to related symptoms. Some cysts may extend to the suprasellar area, leading to hydrocephalus. Headaches often arise from increased dural tension, while visual impairment can occur from optic nerve compression.^5^, ^6^, ^7^, ^8^, ^9^ The T2 sequence enables clear diagnosis and displays the fine structures of the aqueduct of Sylvius and septum. It also allows for the accurate diagnosis and differentiation of suprasellar cisterna arachnoid cysts.^10^, ^11^ In the early stages of the disease, when intracranial pressure differences are minimal, cyst imaging may not be clear. However, as intracranial pressure drops significantly, the difference in pressure can cause cysts to enlarge, thus improving diagnostic clarity. We present four pediatric cases of suprasellar cisterna arachnoid cysts, with the aim of enhancing the diagnosis and treatment of this condition.

## CASE REPORT

**FIGURE 1:**
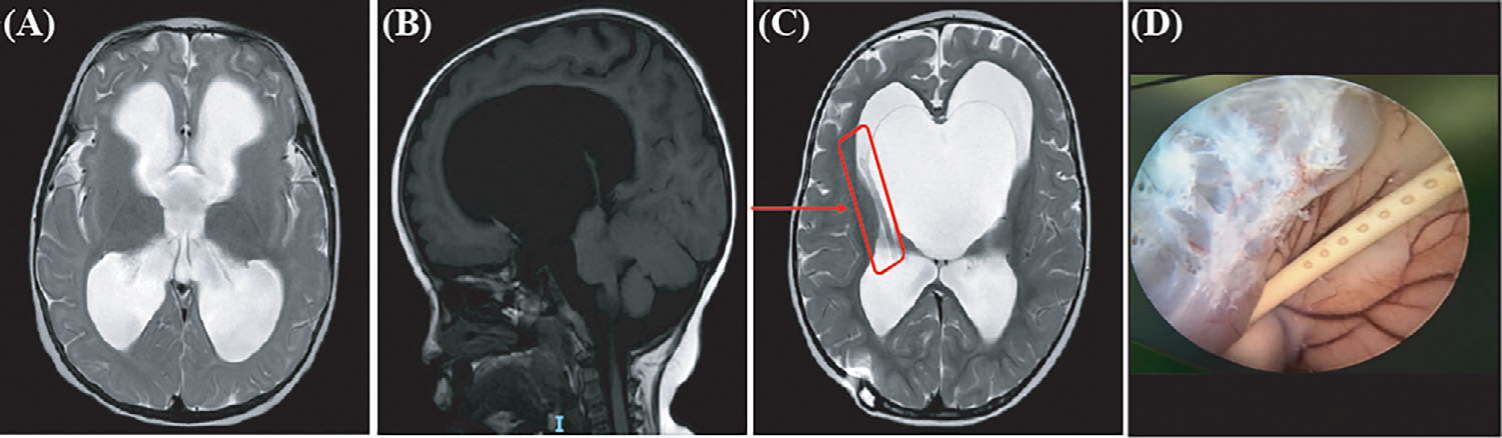
Images of patient 1. (A) Magnetic resonance imaging (MRI)‐T2WI sagittal image and (B) coronal image showing hydrocephalus prior to ventriculoperitoneal shunt surgery; (C) MRI‐T2WI image shows the ventricular tube (red arrow) closely adhering to the ventricular wall, with a large cyst visible; (D) During ventriculostomy, the drainage tubes and cysts are visible.

**Patient 1**: A 5‐year‐old boy was treated for abnormal cranial enlargement noted for over 2 months. He was first admitted on May 25, 2011, and a magnetic resonance imaging (MRI) confirmed the presence of hydrocephalus (Figure 1A, B). A right VP shunt was placed on May 30, using an anti‐siphon, anti‐infection medium‐pressure tube, with regular follow‐ups. On August 5, 2016, computed tomography (CT) showed irregular dilation of the left ventricle. The patient returned on August 9, 2016, complaining of dizziness and headache for over 20 days, which worsened in the last 4 days. Given the presence of a suprasellar cisternal cyst, he underwent a neuroendoscopic third ventriculostomy and cyst fenestration on August 12, 2016. The brain's ventricular tube (red arrow) was visible on the T2 image, closely adhering to the ventricular wall and a large cyst (Figure 1C). During the surgery, a cystic mass was found to block the ventricular foramen. The cyst wall was thick and adhered to brain tissue. The cyst was accessed via ventriculostomy, and the occluded Liliequist membrane was successfully incised and dilated. Drainage tubes and cysts were visible during the ventriculostomy (Figure 1D). No significant bleeding was observed. Pathological examination revealed a fibrous cyst wall with a few cells. Postoperatively, the patient's symptoms improved, and follow‐up was positive.

**FIGURE 2:**
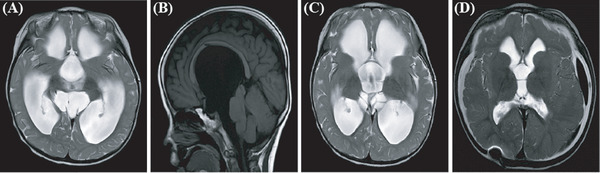
Images of patient 2. (A) Magnetic resonance imaging (MRI)‐T2WI sagittal image and (B) coronal image showing hydrocephalus prior to ventriculoperitoneal shunt surgery; (C) Post‐shunt MRI image showing enlargement of the suprasellar cistern arachnoid cyst; (D) Following ventriculostomy, the suprasellar cistern cyst disappeared, but right subdural effusion and left subdural hematoma were observed.

**Patient 2**: A 3‐year‐old boy was diagnosed with severe hydrocephalus secondary to progressive head enlargement and motor retardation. MRI confirmed severe hydrocephalus, and the patient underwent a right VP shunt in August 2014 (Figure 2A, B). During a reexamination in October 2016, an MRI revealed bulbous dilation of the third ventricle and a suspected suprasellar cistern cyst (Figure 2C). On November 11, 2016, the patient underwent a lateral ventriculostomy and a third ventriculostomy. A giant suprasellar cyst was found in the right ventricle with brain tissue and capillaries on its surface. Bipolar electrocoagulation was used to control bleeding, and a 3 cm opening was made in the cyst wall for pathological examination. Membrane occlusion was observed at the base of the third ventricle. A hole was dissected and expanded to approximately 1.2 cm, with no bleeding. Postoperatively, symptoms improved, and MRI indicated near‐normal ventricular size without hydrocephalus (Figure 2D). Pathological findings revealed a fibrous cyst wall with proliferative small blood vessels and no epithelial covering.

**FIGURE 3:**
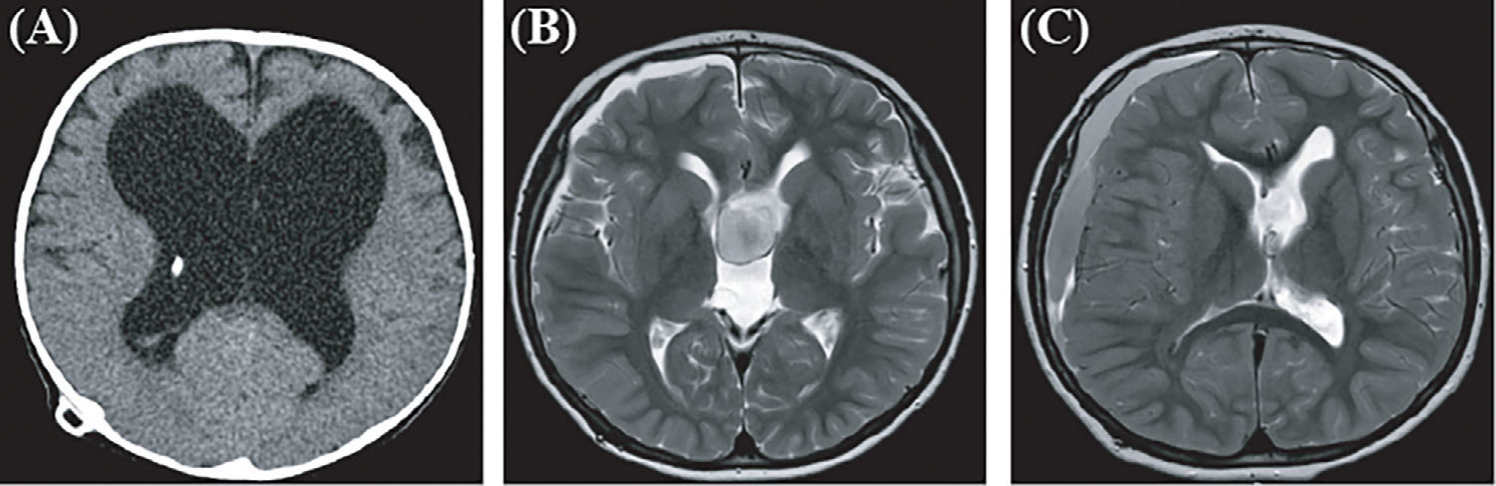
Image of patient 3. (A) Computed tomography (CT) scan showing hydrocephalus with a ventriculoperitoneal shunt; (B) Magnetic resonance imaging (MRI) T2 showing enlargement of the suprasellar cistern arachnoid cyst following shunt placement; (C) Disappearance of the suprasellar cistern cyst after ventriculostomy.

**Patient 3**: A 1‐year‐and‐6‐month‐old male boy was admitted with an abnormal increase in head circumference (52.0 cm) over 2 months. He underwent a lateral VP shunt on January 7, 2020, where the ventricular pressure was measured at a 210 mm water column, and the shunt valve was adjusted to 120 mm (Figure 3A). Two months later, an MRI revealed a suprasellar cistern cyst (Figure 3B). On January 13, 2021, the patient underwent endoscopic third ventriculostomy. During the procedure, a membranous closure was observed at the base of the third ventricle. A hole was created in the vascularized area, expanding it to approximately 0.8 cm in diameter, with no significant bleeding. After ventriculostomy, the suprasellar cistern cyst disappeared, but with left subdural hematoma (Figure 3C).

**FIGURE 4:**
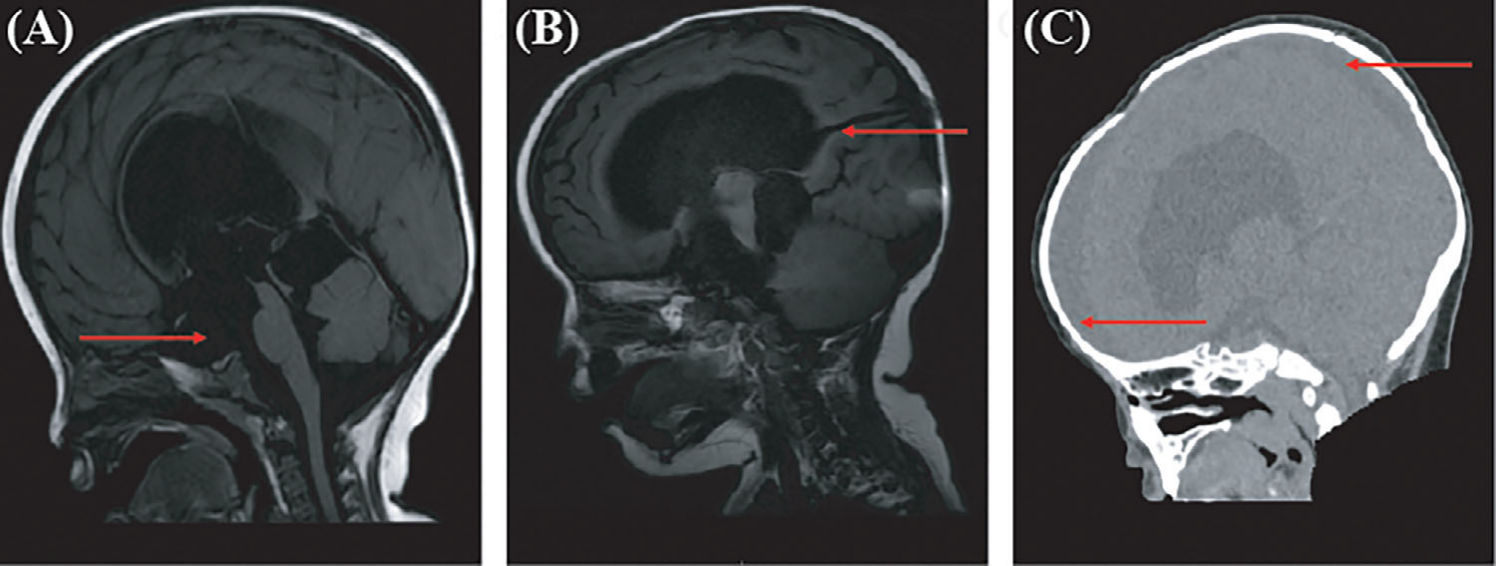
Image of patient 4. (A) Sagittal image magnetic resonance imaging (MRI) showing hydrocephalus prior to ventriculoperitoneal (VP) shunt with a small suprasellar cyst (red arrow); (B) Sagittal image MRI showing hydrocephalus after VP shunt with the ventricular canal shown with a red arrow; (C) After a neuroendoscopic third ventriculostomy and cyst fenestration, subdural effusion was observed in the prefrontal and parietal lobes (red arrow).

**Patient 4**: A 1‐year‐and‐1‐month‐old girl was admitted with progressive head circumference enlargement (52.0 cm) and developmental delay. Sagittal MRI showed hydrocephalus with a small suprasellar cyst (red arrow) (Figure 4A). She underwent a right VP shunt surgery on April 3, 2020. An MRI 2 months post‐surgery revealed a giant suprasellar cisterna cyst filling the ventricle, leading to arachnoid cyst fenestration on May 13, 2020. The ventricular canal (red arrow) was visible in the T1 sagittal image (Figure 4B). A membranous occlusion was noted at the third ventricle, and a 0.8 cm hole was created. Pathology revealed small pockets of parietal tissue with a monolayer of cubic and flattened epithelium (Table S1). Postoperative ventricular dilation was resolved, and subdural effusion was observed in the prefrontal and parietal lobes (red arrow) on sagittal CT (Figure 4C).

## DISCUSSION

Suprasellar arachnoid cysts are typically congenital and primarily affect infants and young children, with low morbidity. The exact pathogenesis remains unclear.^12^ However, several potential causes and mechanisms have been identified: changes in cerebrospinal fluid (CSF) dynamics post‐surgery can lead to fluid accumulation; pressure differences resulting from shunt insertion may promote cyst expansion; inflammatory responses triggered by surgery may encourage cyst growth; shunt malfunctions can exacerbate enlargement; and some arachnoid cysts may grow naturally over time. Understanding these factors is crucial for managing the risk of cyst enlargement after shunt surgery.

The growth of suprasellar arachnoid cysts post‐shunt surgery can lead to a range of complications, including obstructive hydrocephalus, increased intracranial pressure (manifesting as headaches, nausea, and vision issues), and nerve compression (resulting in cognitive changes, seizures, or motor deficits). Significant cyst enlargement may require additional surgery and pose a risk of cyst rupture, which can lead to severe complications and impaired shunt function, thereby hindering CSF drainage. The treatment for symptomatic suprasellar arachnoid cysts remains controversial. Current strategies include regular monitoring, with many advocates of neuroendoscopic transventricular cystomy as an effective method.

In our center, we identified four rare cases of suprasellar arachnoid cysts during follow‐up after VP shunt placement for hydrocephalus. These cases presented with obstructive hydrocephalus symptoms and required a second surgery.^13^, ^14^ In the horizontal imaging position, the ventricle and suprasellar cisterna cyst appear as “Mickey Mouse” signs (Figure 1A). In the early stages, these cysts often show no noticeable symptoms or imaging changes; symptoms and identifiable imaging changes emerge only when significant intracystic pressure differences cause cyst enlargement. Therefore, regular imaging and follow‐up assessments are crucial for effective monitoring.

We speculate that changes in intracranial pressure following shunting affect CSF dynamics, potentially exacerbating existing conditions. Close follow‐up of hydrocephalus patients with brain MRI, especially the three‐dimensional constructive interference in steady‐state sequence, is particularly useful for diagnosing suprasellar cysts. Based on our clinical experience, we recommend follow‐up intervals of 3 months, 6 months, 1 year, 2 years, 3 years, 4 years, and 5 years post‐surgery. If no significant changes were observed within 5 years, the patient's condition was considered stable. However, surgical intervention is required if the symptoms of obstructive hydrocephalus develop.

Our study is currently limited by the small number of patients, but we will continue to gather additional data and follow up on clinical symptoms and imaging findings to further investigate the underlying mechanisms.

## CONSENT FOR PUBLICATION

Written informed consent was obtained from the parents of the patients.

## CONFLICT OF INTEREST

The authors declare no conflict of interest.

## Supporting information



Supporting Information

## References

[1] Liang J , Li K , Luo B , Zhang J , Zhao P , Lu C . Effect comparison of neuroendoscopic vs. craniotomy in the treatment of adult intracranial arachnoid cyst. Front Surg. 2022;9:1054416. DOI: 10.3389/fsurg.2022.1054416 36684173 PMC9852610

[2] Goksu E , Kazan S . Spontaneous shrinkage of a suprasellar arachnoid cyst diagnosed with prenatal sonography and fetal magnetic resonance imaging: case report and review of the literature. Turk Neurosurg. 2015;25:670‐673. DOI: 10.5137/1019-5149.JTN.10655-14.1 26242350

[3] Santiago‐Dieppa DR , Levy ML . Obstructive hydrocephalus. N Engl J Med. 2019;381:e10. DOI: 10.1056/NEJMicm1815973 31365804

[4] Ros‐Sanjuán Á , Iglesias‐Moroño S , Ros‐López B , Rius‐Díaz F , Delgado‐Babiano A , Arráez‐Sánchez MÁ. Quality of life in children with hydrocephalus treated with endoscopic third ventriculostomy. J Neurosurg Pediatr. 2021;27:503‐510. DOI: 10.3171/2020.8.PEDS20384 33607611

[5] Deopujari CE , Kumar A , Karmarkar VS , Biyani NK , Mhatre M , Shah NJ . Pediatric suprasellar lesions. J Pediatr Neurosci. 2011;6:S46‐S55. DOI: 10.4103/1817-1745.85710 22069431 PMC3208925

[6] Massimi L , Bianchi F , Benato A , Frassanito P , Tamburrini G . Ruptured Sylvian arachnoid cysts: an update on a real problem. Childs Nerv Syst. 2023;39:93‐119. DOI: 10.1007/s00381-022-05685-3 36169701 PMC9968703

[7] Ahmad SJ , Zampolin RL , Brook AL , Kobets AJ , Altschul DJ. A case of hydrocephalus confounded by suprasellar arachnoid cyst and concomitant reversible cerebral vasoconstriction syndrome. Surg Neurol Int. 2022;13:331. DOI: 10.25259/SNI_313_2022 36128109 PMC9479517

[8] Julayanont P , Karukote A , Ruthirago D , Panikkath D , Panikkath R . Idiopathic intracranial hypertension: ongoing clinical challenges and future prospects. J Pain Res. 2016;9:87‐99. DOI: 10.2147/JPR.S60633 26929666 PMC4767055

[9] Cao H , Guo G , Wu W , Cheng Z . Classification of the relationship between suprasellar arachnoid cyst and hydrocephalus based on treatment modalities: shunting versus neuroendoscopic approaches. Childs Nerv Syst. 2024;40:2893‐2903. DOI: 10.1007/s00381-024-06478-6 38822205

[10] Li G , Li L , Li Y , Qian Z , Wu F , He Y , et al. An MRI radiomics approach to predict survival and tumour‐infiltrating macrophages in gliomas. Brain. 2022;145:1151‐1161. DOI: 10.1093/brain/awab340 35136934 PMC9050568

[11] Poelmann RE , Gittenberger‐de Groot AC . Hemodynamics in cardiac development. J Cardiovasc Dev Dis. 2018;5:54. DOI: 10.3390/jcdd5040054 30404214 PMC6306789

[12] André A , Zérah M , Roujeau T , Brunelle F , Blauwblomme T , Puget S , et al. Suprasellar arachnoid cysts: toward a new simple classification based on prognosis and treatment modality. Neurosurgery. 2016;78:370‐379. DOI: 10.1227/NEU.0000000000001049. discussion 379‐380. 26445374

[13] Konar S , Nadeem M , Shukla D . Suprasellar multiple neurocysticercal cyst presenting with visual loss. World Neurosurg. 2024;187:67. DOI: 10.1016/j.wneu.2024.04.036 38616022

[14] Donofrio CA , Bertazzoni G , Riccio L , Pinacoli A , Pianta L , Generali D , et al. Intrasellar dermoid cyst: case report of a rare lesion and systematic literature review comparing intrasellar, suprasellar, and parasellar locations. World Neurosurg. 2024;182:83‐90. DOI: 10.1016/j.wneu.2023.11.057 37995988

