# Supplementary material for: Obstruction caused by suprasellar cisterna arachnoid cyst expansion after ventriculoperitoneal shunt in children with hydrocephalus
Source: Pediatr Investig. 2025 Apr 30;9(4):405–9. doi: 10.1002/ped4.70004 (PMC12715886; doi:10.1002/ped4.70004)
Supplement: Supplementary file 1 — Supporting Information [file PED4-9-405-s001.pdf]

Supplementary Materials for

Obstruction caused by suprasellar cisterna arachnoid cyst expansion after ventriculoperitoneal shunt in children with hydrocephalus

Dapeng Li, Wenping Ma, Ming Ge, Di Zhang

Table S1 The detailed information of the four patients

Patient	Gender	Date of VP Shunt	Date of Ventriculostomy	Cyst Pathology
P1	M	05/30/2011	08/12/2016	Cyst with gliosis
P2	M	08/01/2014	11/11/2016	Fibrous tissue, small blood vessels, without epithelial cell
P3	M	01/07/2020	01/13/2021	Dilated vessels, inflammatory cell, without epithelial cells
P4	F	04/03/2020	95/13/2020	Flat epithelium, fibrovascular tissue

P, patient; M, male; F, female; VP shunt, ventriculoperitoneal shunt.
